# The Finer Points of Filopodia

**DOI:** 10.1371/journal.pbio.1000142

**Published:** 2009-06-30

**Authors:** Erik A. Lundquist

**Affiliations:** Department of Molecular Biosciences, University of Kansas, Lawrence, Kansas, United States of America

## Abstract

The nervous system has proven to be a useful model for understanding how cells regulate their shape and motility.

The development of a single-cell zygote into an adult organism depends on highly coordinated, complex processes that control how and when cells divide, move, and change shape. The regulation of cell shape and motility is critical for the formation of functionally distinct tissues and organs and underlies a seminal event during the early development of multicellular organisms called gastrulation—when cells of different tissue types undergo large-scale rearrangements in relation to one another [1]. These processes also play an important role in the adult organism, for example, in wound healing, when fibroblasts and other cells migrate to the site of the injury to begin the process of healing [2], and in pathological situations. For instance, cancer cells migrate from their tissue of origin to populate distinct regions and organs in a process called metastasis [3], which often leads to organ failure and death in cancer patients.

Cells move and change shape at the direction of signals from surrounding tissues, though the molecular mechanisms that drive these signals remain obscure. A new study reported in this issue of *PLOS Biology* sheds light on these processes by describing a novel molecular mechanism that links extracellular signals to cell shape changes in the nervous system [4]. The developing nervous system is a useful model for investigating such mechanisms, because a wide variety of extracellular cues direct neurons as they form the structures and functional connections that make up the central nervous system [5]–[7]. Nascent neurons often migrate from their origin in the lumen of the neural tube to populate distinct distal layers of their target tissues, resulting in the layering of neurons in the spinal cord and cerebral and cerebellar cortices. Neurons must also extend axons to specific regions of the nervous system or periphery to make synapses with the correct partners (e.g., muscles or other neurons), and they remain capable of remodeling throughout adulthood. For example, in the brain, synaptic contacts are dynamically formed, lost, and modified in size and strength in response to neuronal activity, a process referred to as synaptic plasticity [8]. These physical changes in neuronal and synaptic shape are thought to be a basis of learning and memory.

Each of these cell motility events—gastrulation, neuronal migration, axon outgrowth, wound healing, and metastasis—share common cellular features. When observed in the process of development and migration, cells exhibit dynamic extension and retraction of plasma membrane protrusions called lamellipodia and filopodia that are fundamental to cell shape and motility events (Figure 1A) [9],[10]. Lamellipodia (from Latin, “thin plate protrusions”) extend dynamically from the leading edge of migrating cells and axonal growth cones, the specialized structures at the distal tips of developing axons that explore the environment and drive axon extension (Figure 1A). Filopodia (from Latin, “thread protrusions”) also emanate from the leading edges of migrating cells and growth cones, often from the edges of lamellipodia (Figure 1A). Dynamic lamellipodial protrusions are thought to generate the force required for cell and growth cone migration, whereas filopodia are thought to mediate the ability of migrating cells and growth cones to navigate their environments and sense cues as to their direction of migration and destination. Furthermore, filopodia along the shaft of dendrites are thought to be the initiating step in the formation of a new neuronal synapse, a process important in synaptic plasticity, learning, and memory. In this issue of *PLoS Biology*, the Research Article by Menna et al. [4] describes a signaling pathway beginning with an extracellular cue and ending with an actin-binding protein that regulates axonal filopodia formation.

**Figure 1:**
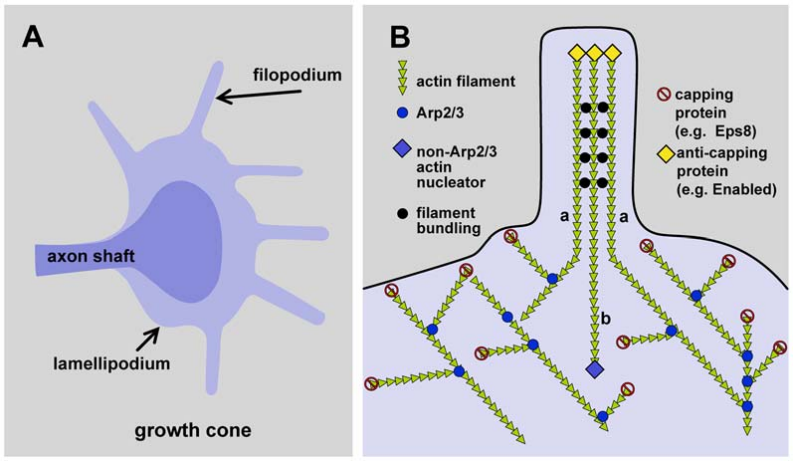
The actin cytoskeleton in lamellipodia and filopodia. (A) Diagram of a growth cone. The axon shaft and growth cone body are represented by dark purple. The thinner lamellipodial and filopodial extensions are indicated by light purple. (B) Schematic diagram of the actin cytoskeleton in a region of lamellipodium containing a filopodium. Actin filaments are indicated by green triangles, with the flat end of the triangle representing the plus or barbed end of the actin filament. The plasma membrane is indicated by a black line. Actin filaments in the lamellipodium are nucleated by Arp2/3 (blue circles) at 70° angles from an existing filament and by non-Arp2/3 factors (blue diamond). Actin filaments are capped by actin-capping proteins, such as Eps8 (red stop signs). Anti-capping proteins, such as Enabled (yellow diamonds), displace capping proteins and allow actin filaments to achieve greater lengths and protrude into filopodial extensions, in which actin filaments are bundled by actin-bundling proteins (black circles).

## Cellular Origins and Functions of Filopodia

It is difficult to discuss filopodial origins without also discussing lamellipodial genesis. The formation of lamellipodia and filopodia is a remarkably complex interaction of multiple cellular components involving coordinated activity of the plasma membrane and the cytoskeleton [9]–[11]. Indeed, the actin cytoskeleton is key to the formation of both lamellipodia and filopodia (Figure 1B). In the lamellipodium, at the leading edge of a migrating cell or growth cone, actin filaments undergo constant polymerization and “push” against the plasma membrane. Actin polymerization is polar, with most polymerization occurring at one end of the actin filament (the plus, or “barbed,” end, so defined by its appearance in electron micrographs after being decorated with the myosin S1 head domain). In leading edges and growth cones, the plus ends of actin filaments are oriented toward the periphery, so most polymerization occurs at the periphery (Figure 1B). At the same time, actin filaments are constantly being pulled away from the periphery via a mechanism involving myosin motor proteins, called retrograde flow. However, peripheral actin filaments are also attached to their substrate through adhesion complexes, such that when myosin motors attempt to drag them away from the periphery, the anchored actin filaments do not move. Instead, plasma membrane and cytoplasm are moved toward the periphery over the anchored actin filaments, resulting in forward translocation. The combination of plus-end actin polymerization, retrograde flow, and cell adhesion in the lamellipodium results in the motile force of cell and growth cone movement.

It has been proposed that a meshwork of actin filaments of variable length and variable parallel orientation to the periphery form the lamellipodium [12],[13]. Filopodia are populated by bundles of long actin filaments that protrude away from the periphery (Figure 1B). Recent studies indicate that the long filopodial actin filaments originate in the lamellipodium [9],[14]. Therefore, one key to the formation of filopodial actin filaments is continued growth at their plus ends. Actin-capping proteins normally block this activity by binding to the plus ends of actin filaments, which stabilizes them and prevents their further growth. A class of proteins called anti-capping proteins, such as the Enabled protein, functionally compete with capping proteins for the plus ends and take their place, thereby allowing the actin filaments to continue plus-end growth [15]. Thus, anti-capping proteins, such as Enabled, are necessary for long filopodial actin filament formation. Indeed, Enabled proteins are localized to the tips (the plus ends) of filopodial actin bundles. Filopodia formation is thought to be dependent, in part, upon the relative activities of actin-capping proteins and anti-capping proteins.

While one role of the lamellipodium seems to be to provide motile force for migration, the role of filopodia is less clear. Enabled, which is required for filopodia formation, is also required for cell and axon outgrowth [16],[17]. However, some migrating cells and growth cones appear to be devoid of filopodia, and, in genetic systems, loss of filopodia (e.g., in an *Enabled* mutant) can have little or no effect on axon outgrowth [18]. One possibility is that filopodia enhance outgrowth and guidance, possibly by exploring the environment for guidance and outgrowth cues. While not absolutely required, filopodia might provide an exploratory function to ensure that the correct cues are found and interpreted. In the nervous system, filopodia that protrude from the lengths of the dendrites might be the initiating events in the formation of post-synaptic structures [19],[20], which specialize in conveying responsiveness to neurotransmitter release. Plasticity of the nervous system depends on the dynamic formation and modification of synapses, and dendritic filopodia might be involved in initiating this process.

## Importance of Localized Filopodia Formation in Response to External Cues

Diverse cues in the extracellular environment—including proteins, carbohydrates, and small molecules—guide migrating cells and growth cones to their targets [21],[22]. Well-characterized examples of guidance cues include proteins of the netrin, slit, and ephrin families. Migrating cells and growth cones express transmembrane receptor molecules that specifically recognize these different guidance cues. In response to these cues, the lamellipodial and filopodial dynamics of the migrating cell or growth cone are altered, resulting in increased protrusion (in the case of an attractive signal) or collapse (in response to a repulsive signal). Thus, extracellular cues influence filopodia formation, and this process is important for guided outgrowth and migration. Likewise, synapse initiation can be controlled by local cues present in the environment. The mechanisms by which extracellular cues are translated into changes in the actin cytoskeleton underlying filopodia formation are not well understood and remain of significant interest to cell and developmental biologists.

**Figure 2:**
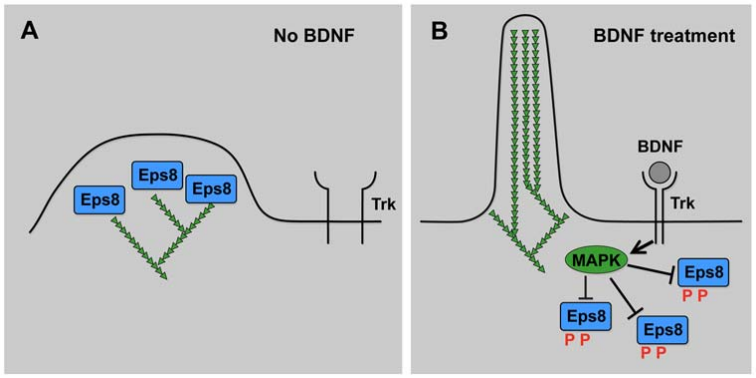
BDNF and MAPK inhibit Eps8, allowing for localized filopodia formation. (A) In the absence of BDNF, Eps8 is an active capping protein that prevents the formation of long filopodial actin filaments. (B) BDNF (gray circle) is detected by Trk receptor tyrosine kinases, which locally activate MAPK signaling. MAPK activation results in phosphorylation (red “P”) and inhibition of the actin-capping activity of Eps8, as well as translocation of Eps8 away from actin-rich peripheral regions. In regions where Eps8 is inactivated, actin filaments are able to achieve greater lengths to mediate the formation of filopodia.

Menna et al. provide insight into this signal transduction process by reporting a mechanism by which an extracellular cue regulates the formation of axonal filopodia in cultured brain hippocampal neurons (Figure 2) [4]. The neurotrophin brain-derived neurotrophic factor (BDNF), a protein secreted by synaptic targets to modulate neuronal survival and differentiation, has long been known to induce axonal filopodia formation when applied to cultured hippocampal neurons [23]. Many other signals as well as cytoplasmic signaling mechanisms that result in actin cytoskeletal change have been identified. What makes this study stand out is that it links BDNF treatment to a specific biochemical activity of an actin-capping protein called Eps8, thus establishing a direct link between extracellular BDNF and axonal filopodia formation. Through genetic analysis, the authors show that Eps8 is normally required to inhibit filopodia formation (Figure 2A). Eps8 has been shown to have multiple effects on actin, including capping activity and actin filament cross-linking activity. Importantly, the authors show that the actin-capping activity of Eps8 is the relevant biochemical activity required to inhibit filopodia formation.

Thus, loss of Eps8 activity had the same consequences as treatment with BDNF, namely enhanced filopodia formation, prompting the authors to ask if BDNF regulated the activity of Eps8 in filopodia formation. BDNF activates Trk receptor tyrosine kinases, which in turn activate MAP kinase signaling inside the cell. Strikingly, they found that phosphorylation of Eps8 in response to BDNF and MAPK inhibited the capping activity of Eps8 and also caused a subcellular redistribution of Eps8 away from actin-rich structures (Figure 2B). Whereas it is clear that molecules that stimulate actin polymerization are required for filopodia formation, this work clearly implicates the inhibition of actin-capping proteins as a key event in filopodia formation. In a growth cone or migrating cell, one can imagine that in response to an external signal (e.g., BDNF), asymmetric deactivation of Eps8 by MAPK on one side of the cell or growth cone could result in asymmetric filopodial protrusion, resulting in altered outgrowth and guidance.

Eps8 is only one of a number of actin-interacting proteins that affect filopodia formation, and further studies will be required to understand how these different molecules work in concert to control filopodia formation in migrating cells and growth cones and in synapse formation. In particular, it will be important to demonstrate that this pathway and others involved in cytoskeletal regulation and filopodia formation have roles in the developing organism, particularly in synapse organization and plasticity. It is likely that this signal transduction pathway involving the anti-capping activity of Eps8 will be involved in other developmental events, such as gastrulation and non-neuronal cell migration, as molecules and signaling pathways often have conserved functions in distinct cell types. Furthermore, this pathway might be important in pathological situations, such as migration of tumor cells during metastasis. While it is possible that BDNF will be the relevant ligand in these cases, it is more likely that Eps8 acts downstream of multiple different ligands in distinct developmental events, reflecting the modularity with which ligands and signaling pathways are iteratively utilized during development. This work on Eps8 in axonal filopodia formation sets the stage for future studies on the role of this pathway in other morphogenetic events in development.
